# Comparative Efficacy of Rectal Diclofenac and Rectal Indomethacin in Preventing Post-endoscopic Retrograde Cholangiopancreatography Pancreatitis: A Systematic Review

**DOI:** 10.7759/cureus.112243

**Published:** 2026-07-07

**Authors:** Shuroog Nabhan, Abdullah Ajwah, Bassam Alsaeedi, Basel Alghamdi

**Affiliations:** 1 Department of Gastroenterology, King Fahad Armed Forces Hospital, Jeddah, SAU; 2 Department of Internal Medicine, King Fahad Armed Forces Hospital, Jeddah, SAU

**Keywords:** endoscopic retrograde cholangiopancreatography, nsaids, post-ercp pancreatitis, prophylaxis, rectal diclofenac, rectal indomethacin

## Abstract

Post-endoscopic retrograde cholangiopancreatography pancreatitis (PEP) is the most frequent complication of endoscopic retrograde cholangiopancreatography (ERCP) and is a major source of ERCP procedure-related morbidity. Nonsteroidal anti-inflammatory drugs (NSAIDs) such as diclofenac and indomethacin are commonly used for PEP prophylaxis because of their anti-inflammatory properties. The aim of the present systematic review was to compare the efficacy of rectal diclofenac versus rectal indomethacin in preventing PEP. The review was conducted using the Preferred Reporting Items for Systematic Reviews and Meta-Analyses (PRISMA) 2020 guidelines, and studies were identified through a comprehensive search of databases covering the period from 2019 to 2025. Nine studies were included. The findings indicate that both rectal diclofenac and rectal indomethacin are effective prophylactic agents and significantly reduce the incidence of PEP. In conclusion, the available evidence supports the routine use of either rectal diclofenac or rectal indomethacin to prevent post-ERCP pancreatitis.

## Introduction and background

Endoscopic retrograde cholangiopancreatography (ERCP) is a complex endoscopic procedure widely used in the evaluation and management of pancreaticobiliary diseases, including choledocholithiasis, biliary strictures, pancreatic duct abnormalities, sphincter of Oddi dysfunction, and malignant biliary obstruction [1]. ERCP has been a revolution in gastroenterological therapy over the past few decades, thanks to its dual use as a diagnostic tool and a minimally invasive therapy. ERCP has several complications, such as hemorrhage, perforation, cholangitis, infection, and pancreatitis, although these complications are clinically not important and therapeutically beneficial. Of these, post-ERCP pancreatitis (PEP) is the most prevalent (and perilous) complication of ERCP. The criteria used to define PEP typically include the development of new or worsening abdominal pain related to an increase in serum pancreatic enzyme levels after ERCP and hospitalization, or an extension of the clinic stay [2,3].

The prevalence of PEP is around 3%-15% in average-risk patients and can be more than 20% in high-risk groups. While most cases are mild and self-constrained, severe cases might also result in pancreatic necrosis, systemic inflammatory response syndrome (SIRS), multiorgan failure, extended hospital stays, higher fitness care charges, and mortality [4]. PEP is thus a significant event for both clinicians and patients undergoing ERCP. PEP is associated with a range of patient- and procedure-associated risk factors. Patient-related factors include female sex, age, sphincter of Oddi dysfunction, a record of pancreatitis, and previous records of PEP [5]. System-related factors include problematic biliary cannulation, pancreatic duct injection, pancreatic sphincterotomy, precut sphincterotomy, and method time [6]. The massive morbidity burden of PEP has brought about extensive research into effective protective processes to ease the prevalence and severity of PEP. Four exclusive prophylactic techniques for preventing PEP were proposed: pancreatic duct stenting, aggressive intravenous hydration, guidewire-assisted cannulation techniques, and pharmacological prophylaxis [7].

Of the pharmacological treatments, rectal nonsteroidal anti-inflammatory drugs (NSAIDs) are among the most effective, low-cost, and convenient preventative treatments. Diclofenac and indomethacin as a rectal preparation are the most common NSAIDs used for PEP prophylaxis [8]. Both agents have been theorized to have anti-inflammatory activity, and their activity has been shown to inhibit the phospholipase A2, the cyclooxygenase-mediated inflammatory pathways, endothelial interaction with neutrophils, and the release of inflammatory cytokines, all of which are involved in the pathogenesis of acute pancreatitis [9]. Their absorptive and pharmacokinetic properties also help to improve their clinical use when administered rectally.

Over the years, several RCTs, observational studies, and meta-analyses have evaluated the efficacy of rectal NSAIDs for PEP prevention. Most of these trials have shown that rectal NSAID administration significantly reduces the incidence and severity of PEP compared with placebo or no prophylaxis [10,11]. So, several international gastroenterology societies, such as the European Society of Gastrointestinal Endoscopy (ESGE) and the American Society for Gastrointestinal Endoscopy (ASGE) recommend the use of rectal NSAIDs routinely as part of preparation for ERCP particularly in those with high risk of developing PEP [12,13]. Diclofenac and indomethacin as rectal preparation have been accepted and are both commonly used as prophylactic drugs; both types of NSAIDs have been found to be effective compared with placebo or no prophylaxis, but the relative effectiveness and safety of the two rectal NSAIDs have not been determined. Some research has indicated that rectal diclofenac may offer better protection against PEP than other drugs due to pharmacodynamic and anti-inflammatory potency differences, whereas other studies have suggested equivalent protection between the two drugs [14,15].

Some discrepancies in the results of the studies may be explained by differences in study design, sample size, patient groups, timing of administration, dosages, procedural techniques, and the definition of PEP used in different studies. Moreover, variations in regional prescribing habits and institutional protocols continue to influence clinical practice when choosing between diclofenac and indomethacin. A detailed comparison of the available evidence between these two rectal NSAIDs is warranted, as the debate is ongoing and evidence varies. Therefore, this systematic review aims to compare the efficacy of rectal diclofenac and rectal indomethacin in preventing post-endoscopic retrograde cholangiopancreatography pancreatitis by systematically reviewing and synthesizing evidence from currently available published studies.

## Review

Methodology

The Preferred Reporting Items for Systematic Reviews and Meta-Analyses (PRISMA) 2020 guidelines [16] were followed to prepare this systematic review. A systematic stepwise approach was used, including the following steps: research question formulation, database search, article screening, eligibility assessment, data extraction, and qualitative synthesis. A systematic search was conducted to identify, screen, and include all relevant studies comparing the efficacy of rectal diclofenac and rectal indomethacin in preventing post-ERCP pancreatitis.

Eligibility criteria

This systematic review was conducted using the PICOTS framework (Population, Intervention, Comparison, Outcome, Timeframe, and Study Design), developed for this review, as shown in Table 1. The PICOTS framework was used to develop the research question and generate precise inclusion and exclusion criteria for studies [17].

**Table 1:** PICOTS format PICOTS: Population, Intervention, Comparison, Outcome, Timeframe, and Study Design; ERCP: endoscopic retrograde cholangiopancreatography; PEP: post-ERCP pancreatitis; NSAIDs: nonsteroidal anti-inflammatory drugs; RCTs: randomized controlled trials.

Structure	Implication	Illustration	Inclusion Criteria	Exclusion Criteria
P	P (Population and/or Patient and/or Problem) Describes the people (or patient group) that the systematic review applies to [18]	Adult patients with ERCP procedures	Adults ≥18 years who have an ERCP performed, male and female participants, human studies	Pediatric patients, pregnant women, animal studies, and patients not undergoing ERCP
I	I (Intervention): The action or factor that is studied or compared. In the present review, prophylactic use of a rectal diclofenac for the prevention of post-ERCP pancreatitis will be included in the intervention group [18]	Pre- or post-ERCP rectal diclofenac administration	Studies evaluating rectal diclofenac administered before or after ERCP for the prevention of post-ERCP pancreatitis	Using oral, intravenous, intramuscular or alternative NSAID routes
C	C (Comparison): The comparison group to assess the effectiveness of the intervention. This can be placebo, conventional treatment, or an alternative prophylactic treatment [18]	Rectal indomethacin or placebo	Studies comparing rectal diclofenac with rectal indomethacin, placebo, or standard care	Studies that lack comparison groups
O	O (Outcome): Clinical outcomes, which are measured to determine the effectiveness of the intervention [18]	Incidence and severity of post-ERCP pancreatitis	Publication reports on incidence, severity, complications, hospitalizations, or adverse events due to PEP	Studies that do not have relevant PEP results
T	T (Time Frame): indicates the time frame or publication period that is relevant to the review [18]	Studies published between 2019 and 2025	Published studies from 2019 to 2025	Studies published before 2019
S	S (Study Design): Strategies for the methodological design of studies that could be included in the review (Randomized controlled trials and observational studies) [18]	Randomized controlled trials and observational studies	Randomized controlled trials, cohort studies, observational studies, systematic reviews, and meta-analyses in English (full text available)	Case reports, non-English articles, Narrative reviews, Letters to the Editor, Editorials, Abstracts of conferences, Unavailable full-text articles

This review was set up as a systematic review with a qualitative synthesis. The main purpose was to compare the ability of rectal diclofenac with that of rectal indomethacin to prevent post-ERCP pancreatitis. The two drugs were not directly compared in the limited studies available, and studies comparing rectal diclofenac or rectal indomethacin with placebo, standard therapy, or no prophylaxis were also found to provide supportive evidence of the effectiveness of each agent. Direct comparative studies were considered primary evidence, and studies with placebo or standard care were considered indirect supportive evidence. Previous systematic reviews and meta-analyses were not pooled with primary studies, but were used as secondary sources of evidence to avoid including the same patients in more than one study, and to prevent overlap.

To validate the research idea of the comparative effectiveness of rectal diclofenac vs rectal indomethacin in the prevention of PEP, a preliminary literature search was conducted on PubMed and Google Scholar. This step was undertaken to prevent duplication of existing reviews, narrow the research question, identify gaps in the current literature, and make the research for the present systematic review feasible.

Databases and search strategy

A sensitive and accurate search method was created using Medical Subject Headings (MeSH) terms, free-text keywords, and Boolean operators. Boolean operators were used to refine the search and to combine or exclude key phrases, including "AND," "OR," and "NOT." The operator "AND" was used to narrow the search by combining different concepts, whereas the operator "OR" was used to broaden the search by including similar or related terms, and the operator "NOT" was used to exclude irrelevant studies [19].

The keywords included in the search were appropriately combined with those required by each database for ERCP, post-ERCP pancreatitis, rectal diclofenac, rectal indomethacin, NSAIDs, and prophylaxis. The included databases and the search strategy are presented in the Appendices. A secondary manual search of the reference lists of selected articles and relevant review studies was additionally performed to identify potentially eligible studies that were not identified in the initial database search. This review includes only human studies published in English between 2019 and 2025 with accessible full texts.

All references retrieved were exported and sorted from the selected databases. The database search was carried out in 2026. Titles and abstracts were then manually and electronically checked for duplicate articles to reduce duplication. Titles, abstracts, and full-text articles were independently screened by two reviewers using the predefined PICOTS inclusion and exclusion criteria. Any disagreement was discussed and, if necessary, a third reviewer was consulted to resolve the disagreement.

No automation tool or machine learning screening software was used in the selection of the studies. Potentially eligible studies were then selected based on the full-text articles, and the inclusion and exclusion criteria were applied. Given that rectal NSAID prophylaxis for post-ERCP pancreatitis is a subject of established significance, the literature search was restricted to studies published from 2019 to 2025, with the goal of generating a synthesis of the latest and clinically relevant evidence.

Quality assessment

The methodological quality of the included studies was evaluated using the Critical Appraisal Skills Programme (CASP) checklist. CASP is a widely used and standardized tool for the critical appraisal of published research that assesses the scientific quality, methodological rigor, credibility, and relevance of research [20]. Each included study was reviewed in several areas, such as clarity of the research question, study design, participant selection, data collection, outcome measurement, ethical issues, validity of the results, and applicability to clinical practice. The quality assessment process was carried out to ensure that the studies included in the final review met methodological standards and provided trustworthy evidence regarding the comparative effectiveness of rectal diclofenac and rectal indomethacin for preventing PEP.

Data extraction

Data extraction was performed systematically using a standardized data extraction form prepared in Microsoft Excel. The relevant data were extracted independently by two reviewers to ensure the accuracy, consistency, and completeness of the information collected from the included studies. The reviewers discussed and agreed on any discrepancies identified during the extraction process.

Data collected comprised the author, year of publication, country of study, study design, sample size, patient characteristics, type and dosage of rectal NSAID used, timing of management, comparator groups, incidence and severity of PEP, adverse events, and the key findings of the study.

The main focus was on outcomes related to the prevention of post-ERCP pancreatitis and on comparing the relative efficacy of rectal diclofenac and rectal indomethacin. Once the data were collected, the co-authors cross-checked them for completeness, consistency, and accuracy and then organized the data into evidence tables for qualitative synthesis and comparison between studies.

 Result

Systematic literature searches were conducted in electronic databases, including PubMed/MEDLINE, Google Scholar, ScienceDirect, Scopus, and Web of Science, and studies that evaluated the comparative efficacy of rectal indomethacin and rectal diclofenac for the prevention of post-ERCP pancreatitis (PEP) were selected. A total of 3265 records were retrieved from the databases. Manual screening of the reference lists of relevant articles and review studies identified additional studies.

After duplicate records were removed, 2523 articles remained for title and abstract screening. Of these, 2410 studies were excluded because they were unrelated to the research question, involved non-ERCP populations, evaluated nonrectal NSAID interventions, were pediatric or animal studies, or were conference abstracts, editorials, or non-English publications. A total of 113 reports were requested and retrieved, whereas 17 requested reports could not be retrieved because the articles were unavailable or the full texts were inaccessible.

**Figure 1:**
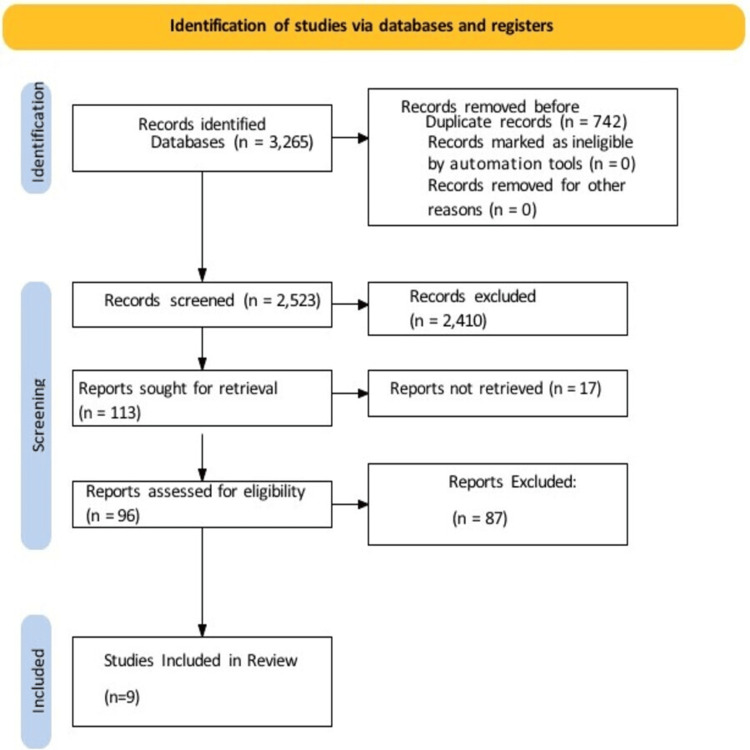
PRISMA 2020 flow diagram PRISMA: Preferred Reporting Items for Systematic Reviews and Meta-Analyses.

Then, 96 full-text articles were evaluated for eligibility based on the inclusion and exclusion criteria. Of these, 87 articles were excluded after full-text assessment. The most common reasons for exclusion were the lack of direct or relevant comparisons between rectal diclofenac and rectal indomethacin, the absence of data from the placebo or standard treatment groups, inappropriate study design, inadequate reporting of PEP-related outcomes, duplicated or overlapping data, inaccessible full text, or poor methodological quality. Finally, nine studies met the eligibility criteria and were included in the qualitative synthesis. The entire study selection process is presented in the PRISMA 2020 flow diagram (Figure 1). 

The selected studies were assessed using the CASP checklist. The quality of all the included studies was good and consistent with the inclusion criteria set for this review. Seven studies achieved a quality score of 10/10, while two studies achieved a score of 9/10 because of minor limitations in the precision of the reported results. The findings from the CASP, however, were interpreted with caution because the evidence included a variety of study designs, including RCTs, retrospective cohort studies, and previous systematic reviews/meta-analyses. The certainty and directness of the evidence were also limited because some studies had small numbers of participants, indirect comparisons, and differing comparators. The CASP scores were therefore taken as supportive evidence for the methodological appraisal but were not considered the sole criterion for the strength of the evidence. All nine studies were thus deemed appropriate for inclusion in the final qualitative synthesis, as shown in Table 2. 

**Table 2:** CASP quality assessment of included studies CASP: Critical Appraisal Skills Programme.

No.	Author and Year	Focused Question	Appropriate Studies	Relevant Studies Included	Quality Assessment Conducted	Appropriate Synthesis	Overall Results Reported	Precision of Results	Applicability	Outcomes Considered	Benefits vs Harms/ Costs	Total Score
1	Fogel et al. (2020) [21]	Yes	Yes	Yes	Yes	Yes	Yes	Yes	Yes	Yes	Yes	10/10
2	Losada et al. (2021) [22]	Yes	Yes	Yes	Yes	Yes	Yes	Can't Tell	Yes	Yes	Yes	9/10
3	Christina et al. (2022) [23]	Yes	Yes	Yes	Yes	Yes	Yes	Yes	Yes	Yes	Yes	10/10
4	Yu et al. (2021) [24]	Yes	Yes	Yes	Yes	Yes	Yes	Yes	Yes	Yes	Yes	10/10
5	Serrano et al. (2019) [25]	Yes	Yes	Yes	Yes	Yes	Yes	Yes	Yes	Yes	Yes	10/10
6	Kang et al. (2022) [26]	Yes	Yes	Yes	Yes	Yes	Yes	Yes	Yes	Yes	Yes	10/10
7	Geraci et al. (2019) [27]	Yes	Yes	Yes	Yes	Yes	Yes	Yes	Yes	Yes	Yes	10/10
8	Abdelfatah et al. (2020) [28]	Yes	Yes	Yes	Yes	Yes	Yes	Can't Tell	Yes	Yes	Yes	9/10
9	Shil et al. (2021) [29]	Yes	Yes	Yes	Yes	Yes	Yes	Yes	Yes	Yes	Yes	10/10

Table 3 summarizes the characteristics and key results of the included studies. The final review included four RCTs, two retrospective cohort studies, and three systematic reviews and meta-analyses. Overall, the evidence showed that rectal indomethacin and rectal diclofenac are effective prophylactic measures against post-ERCP pancreatitis (PEP). Systematic reviews and meta-analyses by Yu et al. (2021) [24], Serrano et al. (2019) [25], and Kang et al. (2022) [26] demonstrated the effectiveness of rectal NSAIDs in the prevention of PEP. Both rectal diclofenac and rectal indomethacin were found to be equally effective by Yu et al. (2021) [24] and Shil et al. (2021) [29], whereas rectal diclofenac was found to be more protective than rectal indomethacin by Kang et al. (2022) [26] and Geraci et al. (2019) [27].

Furthermore, Christina et al. (2022) [23] reported that rectal diclofenac administered before the procedure was a protective factor against the development of PEP compared with administration after the procedure. The evidence is consistent in supporting the use of rectal diclofenac and rectal indomethacin as effective prophylactic treatments for PEP in patients undergoing ERCP, although there are some differences in the results of individual studies. 

**Table 3:** Summary of selected studies evaluating the comparative efficacy of rectal diclofenac and rectal indomethacin in preventing post-ERCP pancreatitis Primary studies were considered the main evidence, while previous systematic reviews and meta-analyses were treated as secondary supportive evidence to avoid overlap or double-counting. ERCP: endoscopic retrograde cholangiopancreatography, PEP: post-endoscopic retrograde cholangiopancreatography pancreatitis, NSAID: nonsteroidal anti-inflammatory drug, RCT: randomized controlled trial, OR: odds ratio, RR: relative risk, RD: risk difference, CI: confidence interval, P: probability value, NNT: number needed to treat, NSTEMI: non-ST-elevation myocardial infarction, TIA: transient ischemic attack, IV: intravenous, IM: intramuscular.

Author/Year	Evidence Type/Role	Study Design and Patient Risk Category	NSAID, Dose and Timing of Administration	Comparator	Main Event Rate/Statistical Findings	Confidence Interval/P-value	Adverse Events	Key Interpretation
Abdelfatah et al. (2020) [28]	Primary study	Retrospective cohort of 2238 ERCP patients (84% low risk, 16% high risk).	Indomethacin 100 mg rectally given at or immediately after ERCP	Rectal indomethacin vs no indomethacin	Overall, PEP occurred in 107/2238 (4.8%). PEP occurred in 48/1055 (4.5%) in the rectal indomethacin group vs 59/1183 (5.0%) in the control group. Rectal indomethacin reduced PEP in consecutive patients by 55% and in low-risk patients by 62%. It also reduced moderate/severe PEP by 47% in consecutive patients and 57% in low-risk patients	Consecutive patients: OR 0.45, 95% CI: 0.36–0.94, P=0.03. Low-risk patients: OR 0.38, 95% CI: 0.19–0.74, P=0.004	No difference in bleeding rate between groups was reported	Rectal indomethacin significantly reduced the incidence and severity of PEP, especially in low-risk patients
Fogel et al. (2020) [21]	Primary study	RCT; 1037 high-risk ERCP patients	Rectal indomethacin: standard dose 100 mg, high dose 200 mg (150 mg immediately after ERCP and 50 mg after 4 hours)	100 mg vs 200 mg rectal indomethacin	PEP occurred in 14.8% (76/515) in the 100 mg group and 12.5% (65/522) in the 200 mg group. Moderate/severe PEP occurred in 5.4% in both groups. Severe PEP was 0.8% vs 0.4%	P=0.32	Clinically significant bleeding: 1.2% vs 1.5%; acute kidney injury: only in high-dose group (0.6%); one NSTEMI in the standard-dose group; one TIA in high-dose group; no deaths or allergic reactions	The rectal 200 mg dose of indomethacin did not significantly improve the results compared with the standard 100 mg dose
Geraci et al. (2019) [27]	Primary study	Randomized double-blinded prospective study; 100 therapeutic ERCP patients; low- and high-risk patients	Diclofenac oral 50 mg, rectal 100 mg suppository, intramuscular 75 mg/3 mL, or intravenous 75 mg/3 mL; administered 30–90 minutes before ERCP	Placebo, oral diclofenac, rectal diclofenac, intramuscular diclofenac, and intravenous diclofenac	Overall, PEP incidence was 11% (11/100). The rectal diclofenac group had 0/20 PEP cases. Placebo: 4/20 (20%); oral diclofenac: 3/20 (15%); intramuscular diclofenac: 3/20 (15%); intravenous diclofenac: 1/20 (5%). Severity overall: 6 mild and 5 moderate cases; no severe pancreatitis reported	P<0.001	Diclofenac was reported to have no adverse reactions in any of the groups	A single 100 mg rectal diclofenac suppository given prior to ERCP was safe and highly effective compared to oral, IM, IV diclofenac, and placebo
Kang et al. (2022) [26]	Secondary supportive evidence	Systematic review and meta-analysis of 26 RCTs; 7954 patients; average- and high-risk patients	Rectal indomethacin or diclofenac ≥100 mg, mostly 100 mg; administered before, during, or after ERCP	Placebo, oral diclofenac, rectal diclofenac, intramuscular diclofenac, and intravenous diclofenac	Overall, PEP incidence was 7.2%. Mild PEP was 5.0%. Moderate/severe PEP was 1.5%. High-risk patients had 8.9% PEP vs 6.4% in average-risk patients. Diclofenac showed lower PEP incidence than indomethacin: 3.8% vs 7.8%. Pre-ERCP NSAID use showed numerically lower PEP than post-ERCP use: 6.1% vs 8.6%	Overall PEP 95% CI: 5.9–8.5%; mild PEP 95% CI: 4.0–6.0%; moderate/severe PEP 95% CI: 0.8–2.3%; diclofenac vs indomethacin P=0.009	There were no significant reports of increased gastrointestinal bleeding or significant NSAID-related toxicity	Standard rectal NSAID prophylaxis reduced severe PEP, and diclofenac may be more protective than indomethacin, although further large direct RCTs are required
Losada et al. (2021) [22]	Primary study	Retrospective cohort; 116 ERCP patients	Rectal diclofenac 100 mg was administered at the beginning of the ERCP procedure	No diclofenac prophylaxis vs rectal diclofenac	Overall, PEP incidence was 8.6% (10/116). PEP occurred in 4 patients without diclofenac and 6 patients with diclofenac. Severe acute pancreatitis occurred in 6 patients, 3 in each group	P=0.196	No mortality and no adverse drug reactions reported	Rectal diclofenac did not significantly reduce PEP in this retrospective cohort
Serrano et al. (2019) [25]	Secondary supportive evidence	Meta-analysis of 21 RCTs; 6854 patients; average and high-risk patients	Rectal diclofenac 50–100 mg or rectal indomethacin 100 mg; predominantly pre- or post-ERCP	Nonsteroidal anti-inflammatory drugs vs placebo/no NSAID	PEP was reduced in the NSAID group: 250/3427 vs 407/3427 in the control group. The mild PEP was drastically reduced. There was no significant decrease reported for moderate or severe PEP. Rectal NSAIDs were the most effective	Overall RD −0.05, 95% CI: −0.07 to −0.03; NNT=20. Mild PEP RD −0.03, 95% CI: −0.05 to −0.01. Rectal NSAIDs RD −0.07, 95% CI: −0.10 to −0.04	No significant increase in the number of adverse events reported across the RCTs included specifically	Rectal diclofenac and indomethacin showed to be the most effective prophylactic NSAIDs, being highly effective against PEP, which was mainly mild
Shil et al. (2021) [29]	Primary study	Prospective, randomized, comparative trial with a retrospective control group of 613 high-risk ERCP patients	Rectal diclofenac suppository 50 mg or rectal indomethacin suppository 100 mg, given during or immediately after ERCP	Diclofenac vs indomethacin vs no NSAID	PEP incidence was 21/247 (8.50%) with diclofenac, 19/244 (7.78%) with indomethacin, and 20/122 (16.39%) with no NSAID. Mild PEP: diclofenac 13 (5.26%), indomethacin 13 (5.33%), no drug 8 (6.56%). Moderate PEP: diclofenac 8 (3.24%), indomethacin 6 (2.46%), no drug 12 (9.84%). No severe pancreatitis reported. Diclofenac and indomethacin showed similar efficacy	Overall P=0.014; diclofenac vs indomethacin P=0.874	No mortality due to PEP reported	No significant difference was seen between these two drugs, and the effect of rectal diclofenac and rectal indomethacin was significantly reduced when compared to no NSAID prophylaxis
Sperna Weiland et al. (2022) [23]	Primary study	RCT; 409 moderate- to high-risk ERCP patients	Diclofenac 100 mg rectally 30 minutes before or after ERCP	Pre-procedure vs post-procedure diclofenac	PEP occurred in 7.5% of the pre-procedure group and 17.5% of the post-procedure group. Mild pancreatitis was significantly lower in the pre-procedure group; no significant difference was reported for moderate/severe pancreatitis	P=0.020	The bleeding rate was 13 cases (2.6%) in the NSAID group as compared to 9 cases (1.8%) in the control group, with few cases of cholangitis and one perforation reported	Pre-procedure rectal diclofenac was statistically more effective than post-procedure administration
Yu et al. (2021) [24]	Secondary supportive evidence	A systematic review and meta-analysis of RCTs of 2928 patients with average risk for ERCP.	Indomethacin 100 mg or Diclofenac 100 mg rectally (usually before ERCP)	NSAIDs compared to no treatment (placebo); diclofenac compared to indomethacin subgroup comparison	Pooled RR for PEP was 0.62 overall. Indomethacin, subgroup RR, 0.67. The RR in the Diclofenac subgroup was 0.42. There was no difference between indomethacin and diclofenac	Overall RR 0.62, 95% CI: 0.46–0.83; indomethacin RR 0.67, 95% CI: 0.49–0.94; diclofenac RR 0.42, 95% CI: 0.23–0.75; diclofenac vs indomethacin RR 1.607, 95% CI: 0.824–3.136	Adverse events occurred in very few cases. The bleeding rate was 13 cases (2.6%) in the NSAID group as compared to 9 cases (1.8%) in the control group, with few cases of cholangitis and one perforation reported	No difference was seen in the efficacy and safety of rectal diclofenac and rectal indomethacin in average-risk patients

Overall, the included studies showed that both rectal diclofenac and rectal indomethacin were effective prophylactic options for reducing post-ERCP pancreatitis. In other studies, such as Shil et al. (2021), direct comparison of diclofenac and indomethacin yielded no significant differences in PEP incidence (8.5%, diclofenac; 7.8%, indomethacin; P=0.874). Some secondary evidence, however, indicated an advantage of diclofenac. For example, Kang et al. (2022) found a lower incidence of PEP in those receiving diclofenac compared with indomethacin (3.8% vs 7.8%, P=0.009). Timing was also suggested to be a factor. In Sperna Weiland et al. (2022), the incidence of PEP was lower when the drug was administered before the procedure (7.5%) than after the procedure (17.5%) (P=0.020). The overall results support the efficacy of rectal NSAID prophylaxis, but the available evidence is insufficient to demonstrate the unambiguous superiority of any single agent.

Discussion

The aim of the present systematic review was to compare the efficacy of rectal diclofenac vs. rectal indomethacin in the prevention of post-endoscopic retrograde cholangiopancreatography pancreatitis (PEP). The findings indicate that both rectal NSAIDs are effective in decreasing the number of PEP cases compared to placebo, standard treatment, or no treatment. Some studies showed a slight superiority in rectal diclofenac, and most evidence indicated a similar efficacy between the two agents; no clear superiority was established. The effectiveness of both drugs is supported by the pathophysiology of PEP, as they have anti-inflammatory effects by inhibiting phospholipase A2 activity, cyclooxygenase-mediated inflammation, cytokine release, and neutrophil activation, thereby decreasing pancreatic injury and preventing clinical progression to pancreatitis. This biological process is consistent with the protective effect observed across all included studies.

Rectal diclofenac or rectal indomethacin versus placebo, usual care, or no prophylaxis was considered only as supportive indirect evidence. These studies have shown that each NSAID is effective on its own compared to the control groups, but have not directly compared diclofenac with indomethacin. Therefore, comparative efficacy was mainly based on studies that directly compared rectal diclofenac with rectal indomethacin, while placebo-controlled or no-prophylaxis studies were used only to support the overall effectiveness of rectal NSAID prophylaxis.

Overall effectiveness of rectal NSAID prophylaxis across the included studies was one of the important findings of this review. In the overall cohort, Abdelfatah et al. 2020 [28] found that indomethacin was effective in preventing PEP in patients by 55% and in low-risk patients by 62%. Similarly, Shil et al. [29] found that 16.39% of patients in the control group had PEP, while 8.50% in the rectal diclofenac group and 7.78% in the rectal indomethacin group had PEP. The results suggest that patients undergoing rectal NSAID prophylaxis have a reduced rate of PEP. This pattern also correlates with previous studies, including that of Elmunzer et al, 2012 [30], which showed some evidence for the use of indomethacin as a prophylactic in high-risk patients. Overall, these findings support the protective effect of rectal NSAIDs in modern ERCP practice.

The issue of relative effectiveness between rectal diclofenac and rectal indomethacin remains the main one examined in this review. Shil et al. (2021) [29] directly compared diclofenac and indomethacin and found no statistically significant difference between the two, although both drugs reduced the incidence of PEP. A meta-analysis by Yu et al. (2021) [24] of 2928 patients showed that diclofenac (RR = 0.42, 95% CI: 0.23-0.75) and indomethacin (RR = 0.67, 95% CI: 0.49-0.94) significantly reduced the risk of PEP. Diclofenac, although showing a numerically lower relative risk, did not indicate any statistically significant difference between the two agents in the indirect comparisons. Results agree with the pioneering study by Alavinejad et al. (2022), who showed that rectal indomethacin significantly reduced PEP in high-risk patients [31]. Overall, the evidence indicates that both rectal diclofenac and rectal indomethacin are clinically effective for PEP and that the current ESGE and ASGE recommendations support their use as PEP prophylaxis.

Although the overall similarity was noted in most studies, some investigations indicated that rectal diclofenac may be beneficial. In the largest meta-analysis in this review (7954 patients), Kang et al. observed a significantly lower pooled incidence of PEP in the diclofenac group than in the indomethacin group (3.8% vs. 7.8%; p = 0.009). Similarly, Geraci et al. showed a significant decrease in PEP after 14 days of rectal diclofenac (0.36; 95% CI: 0.22-0.60). The current results are similar to the previously mentioned randomized study by Sheiybani et al. (2017) [32], which showed a statistically significant decrease in the number of PEP cases after rectal diclofenac administration. The findings are also consistent with earlier evidence reported by Murray et al. (2006) [33], who suggested that diclofenac reduced the incidence of acute pancreatitis following ERCP. The agreement between these studies adds to the evidence of a potentially greater protective effect of diclofenac against pancreatic inflammation. In general, however, it is important to note that most comparisons available are indirect, so it is difficult to draw firm conclusions about the superiority of Diclofenac.

On the other hand, the data presented on the use of indomethacin rectally is just as convincing. Abdelfatah et al. showed a significant decrease in the incidence of PEP in low-risk and all patient groups, and Yu et al. reported a statistically significant decrease in PEP risk in the pooled population of patients receiving indomethacin. In addition, one of the most influential studies in favor of rectal indomethacin prophylaxis is the pivotal study by Elmunzer et al. (2012) [30]. The results provide a rationale for the inclusion of indomethacin in many guidelines and its continued use in clinical practice. As such, the body of evidence in favor of indomethacin should not be overlooked, despite some studies having favored diclofenac.

Some of the studies in this review did not reveal positive results. Losada et al. (2021) [22] found no statistically significant reduction in PEP incidence following rectal administration of diclofenac. The current study, however, was conducted with a small number of subjects (116) and a retrospective design, which could reduce statistical power. Thus, these findings might differ from those of [24, 26, 27] not because of real differences in treatment efficacy, but because of methodological differences. The previous observational studies of PEP prophylaxis have also shown these inconsistencies. Overall evidence still supports the use of rectal NSAID administration, though some studies have failed to support this. Another significant finding from this review is the timing in which it is administered.

Christina et al. (2022) [23] reported that administering rectal diclofenac before ERCP was associated with lower PEP rates than after ERCP. The results corroborate the hypothesis that suppressing inflammatory mediators before tissue damage occurs has the greatest potential for a prophylactic effect. This has been noted in other pharmacokinetic trials, which have demonstrated that rectal NSAIDs reach peak serum levels at the same time as inflammatory activation begins [31]. These indicate that administration time could be significant and could, in part, account for differences in the results of diclofenac and indomethacin studies. Fogel et al. (2020) [21] compared standard dose (100 mg) and high-dose (200 mg) rectal indomethacin in high-risk patients. This is because there was no significant difference between the different doses of NSAIDs, indicating that, after achieving a sufficient level of inhibition of inflammatory pathways, there was no need to increase the dose. These findings are consistent with previous dose-escalation trials and validate the current standard rectal NSAID doses.

The results presented in this review confirm that both rectal diclofenac and rectal indomethacin are effective at decreasing the rate of post-ERCP pancreatitis. These findings are strengthened by consistency across randomized controlled trials, cohort studies, systematic reviews, meta-analysis and international guideline recommendations. Some evidence indicates a slight advantage for rectal diclofenac, especially for the overall incidence of PEP and for administering the drug prior to the procedure, but most evidence shows that these two agents are equally effective. Thus, rectal diclofenac or indomethacin could be recommended as effective prophylactic treatments to lower the burden of post-ERCP pancreatitis, as suggested by the current literature.

Clinical implications

The findings of this systematic review have clinical implications. The evidence consistently suggests that rectal diclofenac and rectal indomethacin are effective prophylactic treatments for post-ERCP pancreatitis. Rectal NSAIDs should be routinely used with ERCP, as this procedure is inexpensive, easy to use, safe, and effective, especially in those who are at risk of PEP. The results indicate that the timing of administration might be important for the prophylactic effect, as preprocedural administration seemed to be more effective than postprocedural administration. Some studies showed a slight benefit of rectal diclofenac, but the overall evidence indicated that either agent could be used. As a result, selection of a specific drug might be based on institutional procedures, accessibility, physician preference, and patient characteristics rather than on significant differences in clinical efficacy.

## Conclusions

The aim of this systematic review was to compare the efficacy of rectal diclofenac and rectal indomethacin for the prevention of post-ERCP pancreatitis. Both agents significantly reduced the occurrence of PEP and proved to be effective prophylactic strategies for patients undergoing ERCP. There was some evidence that rectal diclofenac might have a slight benefit, especially when administered before the procedure, but most of the evidence indicated that diclofenac was as effective as indomethacin.

The consistency of the findings across randomized controlled trials, cohort studies, systematic reviews, and meta-analyses provides greater confidence in the protective effects of rectal NSAIDs. At present, it is not possible to determine which drug is superior. Therefore, rectal diclofenac and rectal indomethacin continue to be evidence-based and clinically useful options for reducing the burden of post-ERCP pancreatitis.

## Appendices

**Table 4:** Search strategy ERCP: endoscopic retrograde cholangiopancreatography, PEP: post-endoscopic retrograde cholangiopancreatography pancreatitis, NSAIDs: nonsteroidal anti-inflammatory drugs, MeSH: Medical Subject Headings, MEDLINE: Medical Literature Analysis and Retrieval System Online.

Databases	Keywords	Search Strategy	Filters	Search Result
PubMed/MEDLINE	Rectal Diclofenac, Rectal Indomethacin, ERCP, Post-ERCP Pancreatitis	((((“Rectal Diclofenac”) OR (“Rectal Indomethacin”)) AND (“Endoscopic Retrograde Cholangiopancreatography” OR ERCP)) AND (“Post-ERCP Pancreatitis” OR Pancreatitis))	Free full text, Humans, English language, Adults ≥18 years, Published 2019-2025	1,024 results
Google Scholar	ERCP AND Rectal Diclofenac OR Rectal Indomethacin AND Pancreatitis	“ERCP” AND (“Rectal Diclofenac” OR “Rectal Indomethacin”) AND “Pancreatitis”	Published in English language; 2019-2025	1,685 results
ScienceDirect	ERCP, Rectal NSAIDs, PEP Prevention	“ERCP” AND (“Rectal Diclofenac” OR “Rectal Indomethacin”) AND (“Post-ERCP Pancreatitis”)	English-language, open-access articles published between 2019 and 2025.	82 results
Scopus	ERCP, Rectal NSAIDs, PEP	(“ERCP”) AND (“Rectal Diclofenac” OR “Rectal Indomethacin”) AND (“Post-ERCP Pancreatitis”)	Human studies, English language, published between 2019 and 2025.	298 results
Web of Science	ERCP, NSAID Prophylaxis, PEP Prevention	(“ERCP”) AND (“Rectal NSAIDs”) AND (“Post-ERCP Pancreatitis”)	Human studies, English language, Published 2019-2025	176 results
